# Air pollution and chronic disease in South Korea’s industrial zones: evidence from a cohort follow-up study

**DOI:** 10.1186/s12889-026-27236-0

**Published:** 2026-06-18

**Authors:** Kil Yong Choi, Ho-Hyun Kim

**Affiliations:** 1 Department of Environmental Energy Engineering, Anyang University, Anyang-Si, Gyeonggi-Do, Republic of Korea; 2 Department of Nano-Chemical, Biological and Environmental Engineering, Seogyeong University, 139, Seogyeong-Ro, Seongbuk-Gu, Seoul, 02717 Republic of Korea

**Keywords:** Pollutants, Emission, Particulate Matter, SAS Enterprise Guide, Industrial complexes

## Abstract

In South Korea, residents living near industrial complexes are subject to environmental pollutant exposure not only from large-scale facilities but also from small businesses. This study investigated the environmental and health impacts of such exposure in Gyeonggi-do using atmospheric dispersion modeling and meteorological analysis. Health effects were assessed through cohort analysis using national health big data and SAS Enterprise Guide. The highest average PM₁₀ concentration over a 3-month period was recorded in 2019 (77.54 μg/m^3^), followed by 2017 (76.32 μg/m^3^) and 2020 (54.02 μg/m^3^). The dominant wind direction was westerly (8%), followed by northwesterly (7%), contributing to pollutant accumulation within a 2-km radius of the industrial area. PM₁₀ levels exceeded the annual average standard (50 μg/m^3^) by 4.0% to 74.8%, corresponding to concentrations of 2.00–37.38 μg/m^3^. Epidemiological analysis revealed a significantly higher incidence of rhinitis in both the exposed (50.31%) and control areas (51.60%) (*p* < 0.01). However, the overall respiratory disease incidence was significantly greater in the exposed group (34.60%) compared to the control group (30.31%) (*p* < 0.01). These results indicate that long-term residence near multipurpose industrial complexes is associated with an increased risk of respiratory diseases and may contribute to cancer and chronic illness. This study provides critical baseline data for future research on environmental health impacts in industrial regions.

**Supplementary Information:**

The online version contains supplementary material available at https://doi.org/10.1186/s12889-026-27236-0.

## Introduction

In Korea, exposure to environmental pollutants from industrial complexes has been shown to affect not only local residents but also small business workers who are not traditionally classified as occupationally exposed individuals [1–3]. Previous studies have reported numerous cases of health deterioration resulting from continuous exposure to various pollutants, with impacts differing depending on the characteristics of each industrial complex. These studies have demonstrated a direct link between such exposures and adverse public health outcomes [4–6]. Air pollution, in particular, has been strongly associated with the development of cancer and chronic diseases through toxicological evaluations [7–9]. Recent evidence also highlights the health risks associated with fine particulate matter (PM₂.₅), indicating that demographic trends, such as population aging, may exacerbate the economic and health burden of PM₂.₅-related diseases [10]. Since the 1970 s, numerous industrial complexes have been established in Yeocheon, Gwangyang, Ulsan, Pohang, and Ansan to promote national economic growth and industrial competitiveness. However, in recent years, these complexes have increasingly been identified as contributors to the rising incidence of chronic diseases and cancers in surrounding communities [11, 12]. Fine particulate matter (PM), often containing heavy metals and chemical substances, has been recognized as a major contributor to air quality degradation, with regional variations based on local industrial and environmental conditions [13, 14]. A comprehensive assessment of air pollution in these regions is urgently needed. For instance, in Ansan-si, even in sectors with relatively low emission ratios, the potential health risks posed by airborne pollutants warrant proactive management of vulnerable populations, particularly those at high risk due to prolonged exposure to low concentrations of toxic substances [15, 16]. According to regional emission inventories from Ansan, air pollutant emissions remain a significant environmental issue, alongside soil contamination. In 2016, air pollutant emissions decreased by approximately 4.78% compared to the previous year. Of the total emissions (1,787,424 kg/year), air pollutants accounted for 1,786,127 kg/year, representing 99.9% of the total. This underscores the dominance of atmospheric discharges. Several prior studies have reported significantly higher prevalence and diagnosis rates of chronic and respiratory diseases in industrial and urban areas with elevated levels of air pollution compared to control areas [17, 18]. These findings suggest that residential exposure to air pollution may contribute to increased rates of chronic and respiratory illnesses among the general population. Environmental exposure to particulate matter has been associated with a wide range of adverse health effects, including asthma, allergic rhinitis, impaired lung function, conjunctivitis, hypertension, atopic dermatitis, cardiovascular diseases, and lung cancer. Accordingly, the present study seeks to address the following research question: Does long-term residential exposure to industrial air pollution—particularly PM₂.₅ and PM₁₀—contribute to a higher incidence of chronic respiratory diseases and cancers among nearby residents compared with control populations? To address this question, we combined atmospheric dispersion modeling with health insurance cohort data in Gyeonggi Province, South Korea, to examine the associations between pollutant exposure and health outcomes.

## Materials and methods

### Hazardous substance emission tracking

Primary data collection in this study involved developing a utilization plan for data managed by the Stack Emission Management System (SEMS). To effectively estimate and convert emission data, additional data processing was required. Specifically, particulate matter (PM) was further classified into chemical components, including organic carbon, inorganic carbon, nitrate, and sulfate. Annual emissions of each component were asessed while accounting for seasonal variability. According to a survey conducted by Gyeonggi Province, the emission source classification system identified detailed subcategories within major source groups, such as manufacturing combustion, production processes, energy transport and storage, waste management, agriculture, and other nonpoint sources [19]. Notably, the emission types and substances recorded in the SEMS may vary depending on these categories. Because self-monitored emissions data are typically collected quarterly or monthly, they may contain errors or inconsistencies. Therefore, it is essential to verify the representativeness of these data through supplementary evaluations. This includes reviewing activity data for each emission source and pollution control facility to ensure accuracy and completeness.

### Air pollution monitoring and measurement methodology

**Fig. 1:**
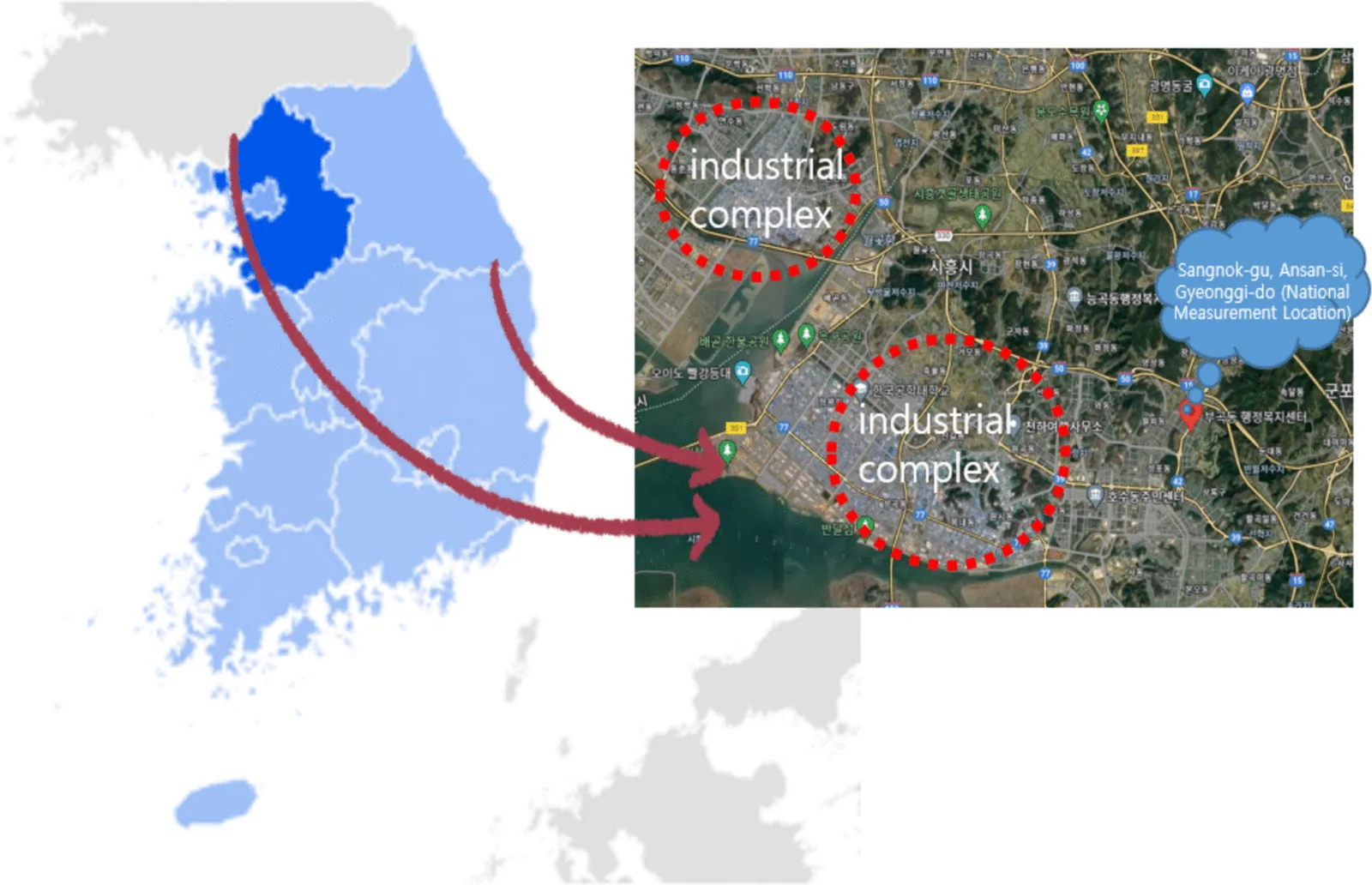
The map of the study area

Air pollution monitoring data used in this study were obtained from the national air quality monitoring network located in Sangnok-gu, Ansan-si, Gyeonggi-do. At this site, air pollutant concentrations are continuously measured and recorded on an hourly basis. For this analysis, both annual average concentrations and 24-h average values of fine particulate matter (PM₁₀ and PM₂.₅) were assessed. According to national standards, the target levels for PM₁₀ are an annual average of 50 μg/m^3^ and a 24-h average of ≤ 100 μg/m^3^. Concentrations were measured using the beta-ray absorption method. The sampling period was determined in accordance with guidelines set by the Ministry of Environment of Korea. Specifically, the period from January to March 2020 was analyzed, as it coincided with the implementation of the seasonal fine dust management policy (“PM₂.₅ Seasonal Control Program”) introduced by the Seoul Metropolitan Government and the Ministry of Environment. The monitoring was carried out over an appropriate duration, based on both internal and external expert consultations, to ensure the representativeness and reliability of the data. In this method, airborne particles with an aerodynamic diameter of 10 μm or less (depending on the separation device used) are collected on a filter medium over a defined time interval. Beta rays are then transmitted through the filter, and the attenuation of these rays is used to determine the mass concentration of particulate matter. The measurement is based on the difference in beta-ray intensity before and after passing through the particle-laden filter, providing a continuous and quantitative estimate of particle mass. The concentration is calculated according to a standardized equation (see Fig. 1). To briefly explain the policy, the seasonal fine dust management policy, known as the “PM₂.₅ Seasonal Control Program,” was jointly implemented by the Ministry of Environment and local governments, including Seoul and Gyeonggi Province. The program designates the winter–spring period (December to March), when fine particulate matter concentrations are typically highest, as a regulatory season. During this period, stricter emission controls are applied to major sources such as industrial facilities, power plants, and vehicles, while supplementary measures, including restrictions on coal heating and enhanced monitoring of vulnerable populations, are also enforced. This policy represents a proactive governmental effort to mitigate the seasonal intensification of PM₂.₅ pollution and to reduce its associated health risks in densely populated urban and industrial regions. Particulate matter concentrations were measured using the beta-ray absorption method, which quantifies dust mass based on the attenuation of beta radiation through a dust-laden filter. The fundamental principle is described by the following exponential relationship:

$$I=Io\times \mathrm{exp}\left(\mu X\right)$$

Where:

I: is the intensity of beta rays passing through the dust collected on the filter paper,

Io: is the intensity of beta rays transmitted through a clean (blank) filter paper,

μ: is the mass attenuation coefficient of beta rays by particulate matter (cm^3^/mg),

X: is the mass of collected dust per unit area (mg/cm^2^).

In this equation, μ is treated as a constant, independent of dust composition. The attenuation of beta rays, therefore, reflects the mass of particles collected per unit area, enabling estimation of airborne particulate concentration.

The dust concentration (C, in mg/m^3^) is calculated using the following formula:

$$C=\left(S/\mu \times V\times \Delta t\right) In \left(I/Io\right)$$

Where:

C: Dust concentration (mg/㎥),

S: is the surface area of the filter where particles are collected (cm^2^),

V: is the volume of sampled air (m^3^),

Δt: is the sampling duration (minutes),

ln(I/I₀): the natural logarithm of the beta-ray intensity ratio.

This method provides a continuous and quantitative assessment of airborne particulate concentrations and is widely adopted in environmental monitoring networks due to its reliability and precision.

Data used in this study were obtained from the National Ambient Air Quality Monitoring Information System (NAMIS), which collects real-time air quality data through an internet-based network. Monitoring stations located in Seoul, Gyeonggi-do, and Incheon provided concentration data for fine particulate matter (PM). To ensure accurate particle size separation and concentration measurement, air samplers were operated at a constant flow rate of 16.7 L/min. If the sampling flow deviated by ± 10%—specifically, dropping below 15.0 L/min or exceeding 18.4 L/min—for more than one minute, the sampling process was immediately halted, and the equipment was inspected and recalibrated. The monitoring network ensures data reliability through adherence to national standardized protocols and automated quality control procedures. Only data meeting these operational standards were used as the basis for the air pollution analysis in this study.

### Utilization of surrounding weather and atmospheric models

In contrast, PM₂.₅ concentrations consistently exceeded the national annual standard (15 μg/m^3^) across all monitored locations, including Jeongwang-dong, Wongok-dong, the Gyeonggi-do average, the national average, and the average for major cities—indicating a more persistent and widespread issue with fine particulate pollution. To simulate the atmospheric dispersion of pollutants, the CALPUFF modeling system was used. The modeling domain incorporated both meteorological and dispersion components. High-resolution, three-dimensional meteorological fields were generated using a nested grid model, and the final spatial resolution of 0.2 km was converted into input data for the CALMET module [20]. This enabled realistic simulation of meteorological conditions affecting pollutant transport. Topographical data with a spatial resolution of 1 arc-second (approximately 30 m) and land-use classification data from the Ministry of Environment were used to accurately represent surface characteristics within the CALMET model domain.

### Establishment and analysis of a cohort based on health big data

Cancer incidence data were obtained from multiple sources, including the Gyeonggi-do Cancer Registration Headquarters, early cancer screening programs, and individual hospital records accessed via the National Cancer Center [21]. These data enabled comprehensive analyses of cancer patterns by integrating both registry and publicly available datasets. Mortality data were sourced from the Social Statistics Bureau of the National Statistical Office and local district offices. Unlike disease prevalence data, mortality data are considered more accurate and reliable, as death is a clearly defined, single-occurrence event that can be objectively verified [22]. Death statistics—part of vital statistics (which also include birth, marriage, and divorce)—are collected through a mandatory notification system. Deaths must be reported to the relevant authorities within one month of occurrence, and causes of death are classified as direct, intermediate, or antecedent. Customized health data were extracted from the National Health Insurance Service (NHIS) Data Sharing Service (https://nhiss.nhis.or.kr) [23]. The NHIS dataset includes insurance claims data, diagnostic codes, treatment details, and prescription records. It is structured into three primary databases: the eligibility database (demographic information such as gender, age, residence, and income), the treatment database (including diagnosis codes and service records), and the health screening database. This comprehensive resource supports long-term prospective cohort studies for evaluating chronic disease outcomes. For this study, all individuals whose residential codes indicated residence within either the Gyeonggi-do industrial complex exposure area or the designated control area between 2007 and 2019 were included. Data from 2002 to 2006 were excluded due to instability in residence information. The constructed cohort dataset enabled continuous follow-up based on the duration of residence and facilitated comparisons of chronic disease and cancer incidence between the exposed and control populations. Statistical analyses were performed using SAS Enterprise Guide and SAS software. A Cox proportional hazards model was employed to estimate hazard ratios (HRs) for the development of chronic diseases according to residential area. Both crude models and models adjusted for age, sex, and income were used to evaluate associations across 23 chronic disease categories.

## Results

### Results of particulate matter concentration in industrial complexes

Analysis of PM₁₀ data from national industrial complexes revealed discrepancies between emission quantities and ambient concentration levels. The highest quarterly average PM₁₀ concentration was recorded in 2019 at 77.54 μg/m^3^, followed by 76.32 μg/m^3^ in 2017 and 54.02 μg/m^3^ in 2020 (Table 1). Despite fluctuations in emissions, ambient PM₁₀ concentrations did not always correlate directly with the volume of emissions generated. This study focused on January–March 2020, during the implementation of the seasonal fine dust management policy by the Seoul Metropolitan Government and the Ministry of Environment. Although monitoring data from other years were limited, the availability of 2020 data provided an opportunity to evaluate the effectiveness of this policy framework. Accordingly, the analysis focused on this designated management period to establish an evidence base for policy recommendations aimed at improving air quality and mitigating health risks associated with fine particulate matter. These findings suggest that factors beyond emission quantity—such as meteorological conditions, atmospheric dispersion patterns, and local topography—may significantly influence ambient PM₁₀ levels. The observed decline in both emissions and ambient concentrations in recent years highlights the potential effectiveness of South Korea’s particulate matter (PM) management policies and underscores their role in improving air quality (Table 1).

**Table 1:** Average, standard deviation, min, and max analysis results of air pollution concentration by Particulate Matter

Date	N (Daily average)	Average	Standard Deviation	Min	max
Jan ~ Mar 2020 PM_10_	90	**54.02**	**22.05**	**4**	**103**
Jan ~ Mar 2019 PM_10_	90	**77.54**	**38.94**	**29**	**216**
Jan ~ Mar 2017 PM_10_	90	**76.32**	**26.33**	**32**	**164**
Apr ~ Jun 2020 PM_10_	91	**39.08**	**17.14**	**7**	**118**
Jul ~ Sept 2020 PM_10_	92	**22.71**	**11.80**	**2**	**59**
Oct ~ Dec 2020 PM_10_	92	**45.84**	**21.03**	**13**	**119**

### Execution of the model (CALPUFF) for the surrounding area in Ansan

**Fig. 2:**
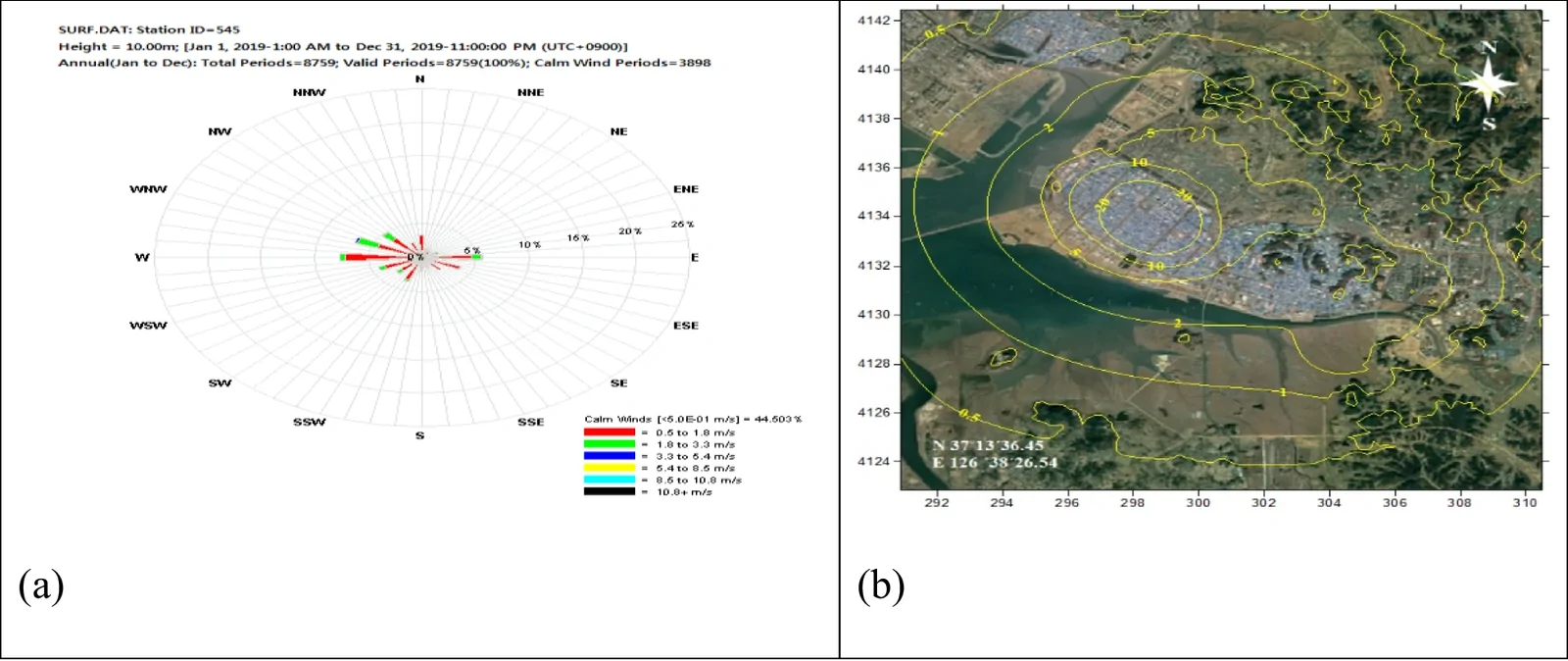
Average annual wind rose and pollutant diffusion simulation results (**a**: wind rose, **b**: PM10)

Atmospheric dispersion modeling was performed using emission estimates from the target industrial area. Backward trajectory analysis, incorporating annual wind rose data and pollutant diffusion simulations, was conducted to predict PM₁₀ dispersion for the year 2019. The predominant wind directions were westerly (approximately 8%) and northwesterly (approximately 7%), with the highest wind speeds (3.3 to 5.4 m/s) observed in the northwesterly direction. Overall, wind patterns were relatively evenly distributed throughout the year. The pollutant dispersion model revealed a relatively uniform spread of PM₁₀ across the surrounding area, driven by prevailing wind conditions. The primary downwind impact zones included adjacent urban regions such as Siheung-si and several districts within Ansan-si, including Wongok-dong, Choji-dong, and Singil-dong. Within a 2-km radius of the emission source, annual PM₁₀ concentrations ranged from 2.00 to 37.38 μg/m^3^, corresponding to 4.0% to 74.8% of the national annual air quality standard (50 μg/m^3^) (Fig. 2). These results indicate that communities located near the industrial complex may experience elevated exposure levels depending on proximity to emission sources and meteorological conditions.

**Fig. 3:**
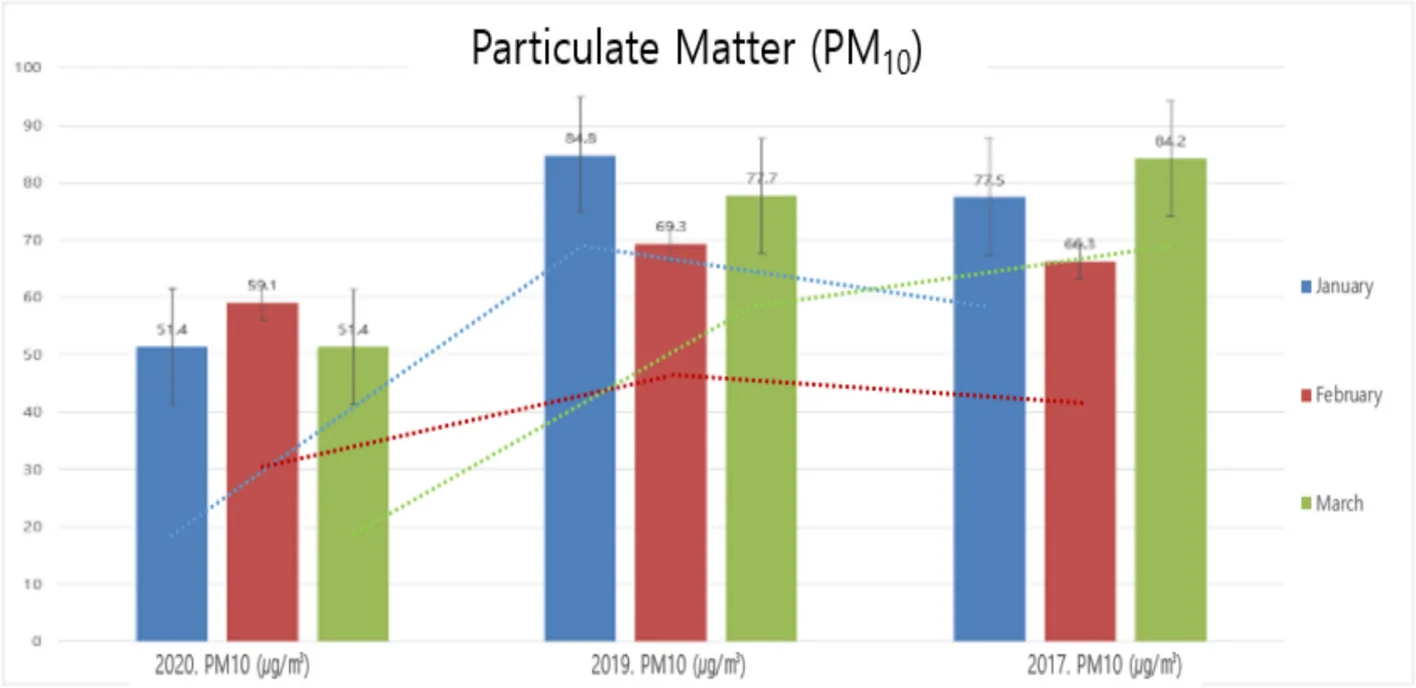
Concentration characteristics for the Particulate Matter policy period (January to March)

In South Korea, analysis of emission data during the designated fine dust management period (January to March) revealed that reductions in particulate matter (PM) emissions were not effectively achieved. Despite the implementation of national-level policies aimed at controlling fine particulate matter, no significant decrease in PM concentrations was observed in industrial areas during this period. This finding was statistically significant when comparing data from 2017—a year marked by elevated emissions—with that of 2019, indicating that emission levels remained inadequately controlled despite policy interventions (Fig. 3). These results highlight the need to enhance long-term air quality management strategies, particularly in industrial complexes where elevated PM concentrations may persist regardless of short-term regulatory efforts. Future mitigation approaches should incorporate both temporal and spatial emission patterns to more effectively address chronic exposure risks associated with industrial emissions.

This study focused on January–March 2020, during the implementation of the seasonal fine dust management policy by the Seoul Metropolitan Government and the Ministry of Environment. Although monitoring data from other years were limited, the availability of 2020 data provided an opportunity to evaluate the effectiveness of this policy framework. Accordingly, the analysis focused on this designated management period to establish an evidence base for policy recommendations aimed at improving air quality and mitigating health risks associated with fine particulate matter.

Particulate matter (PM₁₀) concentrations were notably elevated in both 2017 and 2019. In particular, 2017 exhibited high ambient concentrations coinciding with elevated emission levels. Using 2017 as the reference year, interannual variations in PM₁₀ concentrations were statistically evaluated. Although the average PM₁₀ concentration in 2020 (67.70 μg/m^3^) appeared higher than that in 2017 (52.23 μg/m^3^), the difference was statistically significant (*P* = 0.0141), indicating a meaningful temporal fluctuation. However, when data were stratified by quarter, the elevated concentrations observed during April to June 2020 did not reach statistical significance compared to other periods (Table 2).

**Table 2:** Correlation analysis of concentrations by year of Particulate Matter

PM10(ppm)	Mean(N)	95% CL Mean		*P*-value
Jan ~ Mar 2017 PM10	52.23	45.35	59.10	0.8627
Jan ~ Mar 2019 PM10	46.26	40.26	52.26	
Jan ~ Mar 2020 PM10	67.70	58.17	77.23	0.0141
Apr ~ Jun 2020 PM10	55.91	49.44	62.39	0.4337
Jul ~ Sept 2020 PM10	38.98	33.85	44.11	0.8413
Oct ~ Dec 2020 PM10	22.37	18.89	25.85	0.6739

**Fig. 4:**
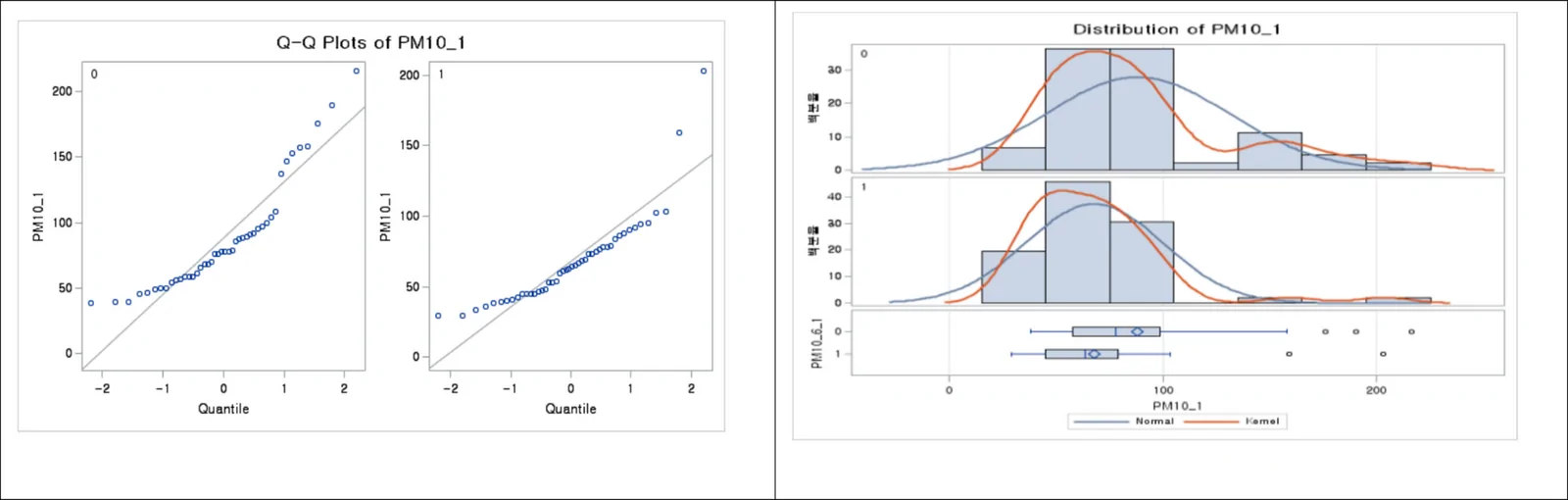
Relationship between the percentage of concentrations by year of Particulate Matter

**Fig. 5:**
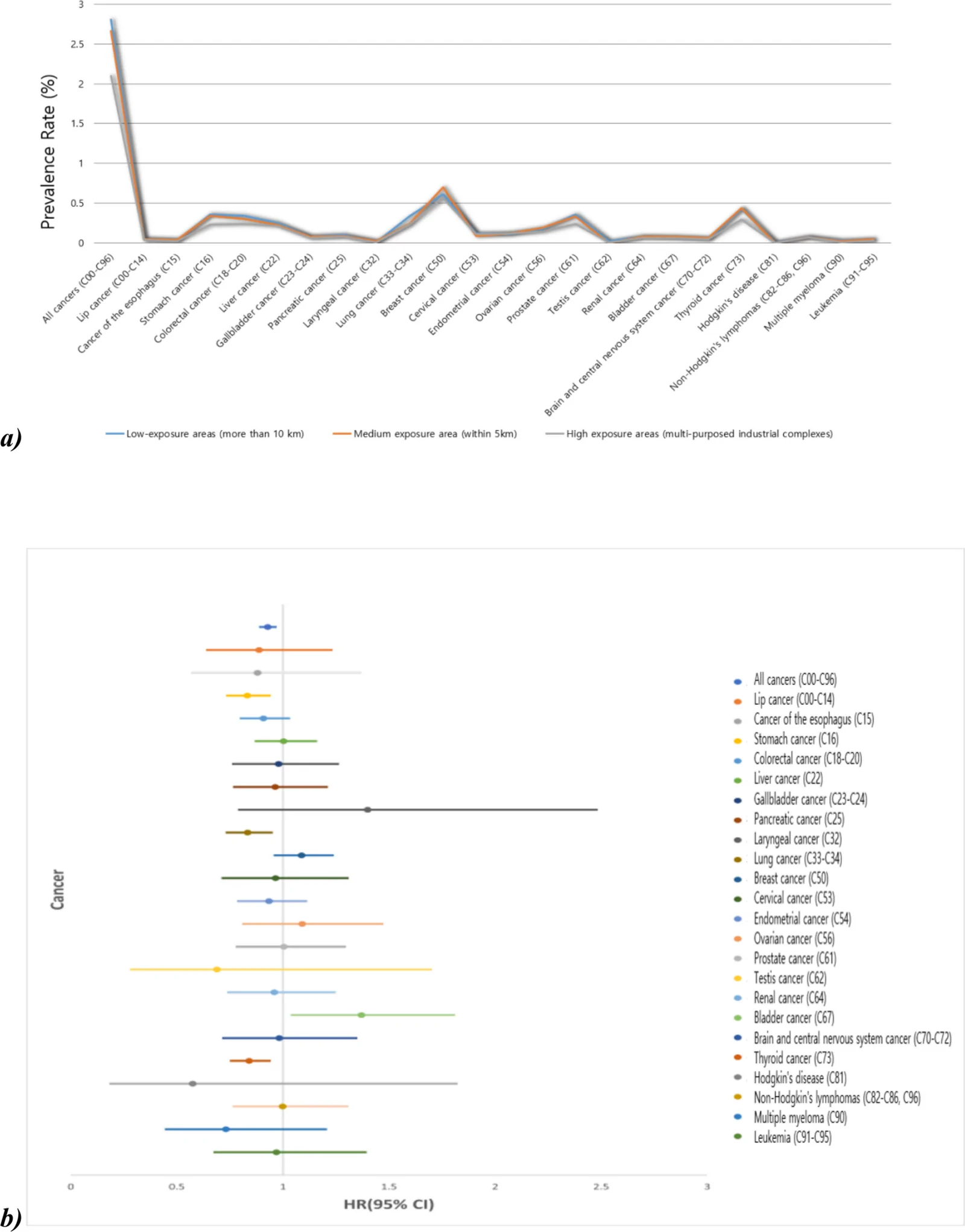
Cancer incidence by exposure to environmental pollution among cohort subjects. **a** Trends in cancer incidence across 24 types of cancer in the exposed and control (medium and low exposure) regions. **b** Comparative analysis of cancer incidence in the exposed and control areas for residents living for more than 10 years

The relationship between annual changes in PM₁₀ concentrations and emission levels was further supported by statistical trend analysis (Fig. 4), with the association visually illustrated through a correlation plot (Fig. 5). These findings suggest that temporal variations in PM₁₀ concentrations may reflect broader shifts in emission dynamics, warranting further investigation into the underlying factors driving year-to-year variability.

### The status of incidence of meniscus cohort subjects by chronic disease.

In this study, residents of Banwol-dong were designated as the exposure group, while those living in Jeongwangbon-dong, Jeongwang-dong, Wongokbon-dong, Wongok-dong, Choji-dong, and Gojan-dong were classified as the control group. According to the Ministry of Environment, areas within 2–5 km of the industrial complex are defined as exposure zones, whereas regions outside this range are considered controls for environmental management and compensation purposes. This classification allowed the analysis to examine overlapping high-risk characteristics during the PM₂.₅ Seasonal Control Program, providing insight into environmental factors influencing vulnerable populations. A total of 454,612 individuals were included in the study cohort, comprising 171,300 residents from the exposed area (Banwol-dong) and 283,312 residents from the control areas (Jeongwangbon-dong, Jeongwang-dong, Wongokbon-dong, Wongok-dong, Choji-dong, and Gojan-dong). In the exposed group, the gender distribution was 53.48% male (*n* = 91,614) and 46.52% female (*n* = 79,686), while the control group consisted of 50.50% male (*n* = 143,066) and 49.50% female (*n* = 140,246). The age distribution in the exposed area was as follows: 33.69% (*n* = 57,715) aged 20–39 years, 32.44% (*n* = 55,573) aged 40–64 years, 28.83% (*n* = 49,378) under 20 years, and 5.04% (*n* = 8,634) aged 65 years or older. In comparison, the control area included 35.57% (*n* = 100,788) aged 40–64 years, 30.08% (*n* = 85,218) under 20 years, 26.84% (*n* = 76,049) aged 20–39 years, and 7.50% (*n* = 21,257) aged 65 years or older. In terms of income level, individuals in the third income quintile represented the largest proportion in both groups—35.01% in the exposed area and 31.88% in the control area. The mean follow-up duration was 8.49 ± 3.83 years for individuals in the exposed group and 9.03 ± 3.62 years in the control group. The total person-years observed were 1,385,318.91 in the exposed group and 2,439,335.40 in the control group (Table 3).

**Table 3:** General characteristics of all subjects according to the half-winter exposure area and control area

-		Exposure area	Control area	*p*-value*
Total (persons)		171,300	283,312	
Gender	Male	91,614(53.48)	143,066(50.50)	0.0001
	Formal	79,686(46.52)	140,246(49.50)	
Age (years)		33.05 ± 18.11	34.47 ± 19.73	-
	< 20	49,378(28.83)	85,218(30.08)	0.0001
	20–40	57,715(33.69)	76,049(26.84)	
	40–65	55,573(32.44)	100,788(35.57)	
	≥ 65	8,634(5.04)	21,257(7.50)	
Income**	1 st quartile	28,074(16.49)	50,659(18.08)	0.0001
	2 st quartile	38,375(22.55)	59,445(21.22)	
	3 st quartile	59,589(35.01)	89,320(31.88)	
	4 st quartile	44,175(25.95)	80,742(28.82)	
Average observation period (years)		8.49 ± 3.83	9.03 ± 3.62	-
Total Observed Years (years)		1,385,318.91	2,439,335.40	-

^*^Chi-square test

^**^Income decile: Health insurance premium 20 deciles divided into quartiles

The incidence of representative respiratory diseases was evaluated to assess the exposure–response relationship with environmental pollution in Korea. Incidence rates were calculated as average incidence densities, defined as the number of new cases divided by total person-years of observation. Among respiratory illnesses, the incidence of acute upper respiratory tract infections was significantly higher in the exposed area compared to the control area (15,189 cases [57.05%] vs. 23,961 cases [52.83%], *p* < 0.01). No statistically significant difference was observed in the incidence of other upper respiratory tract diseases between the exposed and control areas (74,950 cases [74.58%] vs. 128,434 cases [74.71%], *p* = 0.4510). Conversely, a significant difference was found in the incidence of other acute lower respiratory tract infections, with higher rates in the exposed area (25,429 cases [47.03%]) than in the control area (36,108 cases [43.17%], *p* < 0.01). Asthma incidence was also significantly elevated in the exposed population compared to the control group (129,659 cases [93.87%] vs. 200,437 cases [90.57%], *p* < 0.01) (Table 4).

**Table 4:** Status of Respiratory Diseases in Meniscus (Unit, No. (%)

**-**		**Exposure area**	**Control area**	*p*-value*
**Acute upper respiratory tract disease(J00-J06)**				
Number of subjects (persons)		26,625	45,353	
Result of occurrence	Yes	15,189(57.05)	23,961(52.83)	<0.0001
	No	11,436(42.95)	21,392(47.17)	
Other upper respiratory tract diseases (J32-J39)				
Number of subjects (persons)		100,501	171,918	
Result of occurrence	Yes	74,950(74.58)	128,434(74.71)	N.S.
	No	25,551(25.42)	43,484(25.29)	
Other acute lower respiratory tract infections (J20-J22)				
Number of subjects (persons)		54,071	83,644	
Result of occurrence	Yes	25,429(47.03)	36,108(43.17)	<0.0001
	No	28,642(52.97)	47,536(56.83)	
Asthma (J45-J46)				
Number of subjects (persons)		138,125	221,294	
Result of occurrence	Yes	129,659(93.87)	200,437(90.57)	<0.0001
	No	8,466(6.13)	20,857(9.43)	

Among other respiratory conditions, the incidence of rhinitis differed significantly between the exposed and control groups, with 30,158 cases (50.31%) reported in the exposed area and 50,602 cases (51.60%) in the control area (p < 0.01). The overall incidence of respiratory diseases was also significantly higher in the exposed area, with 5,442 individuals affected (34.60%) compared to 7,292 individuals (30.31%) in the control area (p < 0.01). Additionally, the incidence of cough was markedly higher in the exposed population, with 163,864 individuals (97.70%) reporting symptoms versus 265,035 individuals (96.00%) in the control population (p < 0.01) (Table 5).

**Table 5:** Comparison of rhinitis, respiratory diseases, and cough between exposure and control areas (Unit, No. (%)

**-**		**Exposure area**	**Control area**	*p*-value*
**Rhinitis (J30-J31)**				
Number of subjects (persons)		59,946	98,060	
Result of occurrence	Yes	30,158(50.31)	50,602(51.60)	<0.0001
	No	29,788(49.69)	47,458(48.40)	
Respiratory disease (J00-J99)				
Number of subjects (persons)		15,730	24,062	
Result of occurrence	Yes	5,442(34.60)	7,292(30.31)	<0.0001
	No	10,288(65.40)	16,770(69.69)	
Cough (R05)				
Number of subjects (persons)		16,7726	276,088	
Result of occurrence	Yes	163,864(97.70)	265,035(96.00)	<0.0001
	No	3,862(2.30)	11,053(4.00)	

### Current status of cancer incidence among cohort subjects of multi-purpose industrial (centered on Sihwa Banwol Industrial Complex)

Cancer incidence in multi-purpose industrial areas was analyzed by categorizing the control area into medium- and low-exposure zones, defined as populations residing 5 km and 10 km or more away from the industrial complexes, respectively. The exposure area included individuals living within 2 km of the industrial complex. Risk factors were identified based on residential history, focusing on multi-purpose industrial workers in Korea who had resided in these areas for over 10 years, accounting for the typical incubation period of cancer development. Analysis of total cancer incidence revealed a significant difference between the exposure and control regions (p < 0.05), suggesting that cancer and chronic disease prevalence increase in correlation with the severity of environmental pollution experienced by residents living near industrial complexes for more than a decade. Specifically, the incidence rates of stomach cancer, colorectal cancer, and prostate cancer were 0.34%, 0.30%, and 0.34%, respectively, in the exposed area—significantly higher than the rates observed in the control area (0.22%, 0.24%, and 0.24%; p < 0.0001). In contrast, the incidence of esophageal cancer was lower in the exposed area (0.02%) compared to the control area (0.03%), and this difference was statistically significant (p < 0.05). Meanwhile, lung cancer (0.25%) and breast cancer (0.55%) exhibited notably higher incidence rates than other cancers, with these differences also reaching statistical significance. These findings underscore the critical importance of managing environmental exposures as key risk factors for cancer and chronic diseases. Although the overall prevalence of environmental risk factors in Korea often remains below 0.2%, the elevated incidence of lung and breast cancers highlights the necessity for ongoing management and preventive strategies targeting exposure to atmospheric pollutants, which are recognized as primary contributors to cancer development (Fig. 5a,b).

## Discussion

This study examined the characteristics of air pollutant emissions and their potential associations with chronic respiratory diseases in an industrial area of South Korea. While the emission volumes fluctuated over the study period, the consistently elevated levels of particulate matter and chemical emissions indicate continued exposure risks for nearby residents. The observed pollution trends generally align with previous reports from Korean industrial complexes, suggesting that industrial activities remain an important contributor to local air quality conditions. The accumulated data provide valuable insights into the relationship/association between environmental pollution and health outcomes [24]. Notably, 2017 exhibited the highest overall trend in pollutant discharge. Analysis of chemical emissions according to the management plan for inducers—including air emissions (kg/year), total emissions (kg/year), and movement amounts (kg/year)—also peaked in 2017. Yearly follow-up data from 2016 to 2020 reported air emissions of 1,963,762; 1,800,779; 1,938,884; 2,136,079; and 1,786,127 kg/year, respectively. Similarly, total emissions were recorded as 1,963,954; 1,801,337; 1,939,663; 2,136,219; and 1,787,424 kg/year, respectively. The data confirmed that emissions in 2020 were higher than in 2019, reflecting the characteristics and dynamics of emitted substances. Using dispersion modeling and meteorological analyses, this study suggested that pollutants were likely transported toward nearby residential areas under prevailing wind patterns. Although the exposure estimates were based on annual averages rather than continuous measurements, the spatial patterns were consistent with known industrial emission sources. The contribution of environmental pollution to health was further analyzed using a regional atmospheric diffusion model [25]. The study identified chronic disease characteristics associated with pollution, showing that pollution sources remained high even in the absence of external factors [26]. The highest average air pollution concentration over a three-month period was recorded in 2019 at 77.54 μg/m^3^, followed by 76.32 μg/m^3^ in 2017 and 54.02 μg/m^3^ in 2020. Despite these elevated pollution levels, a direct impact on health outcomes could not be confirmed, after accounting for individual characteristics and external factors such as COVID-19, which may have influenced health independently. Further investigation of pollution sources was conducted using detailed measurement data and meteorological information, including external influences [27]. Meteorological analysis revealed that the dominant wind direction was westerly (~ 8%), followed by northwesterly (~ 7%), with maximum wind speeds ranging from 3.3 to 5.4 m/s in the northwesterly direction, and a relatively even distribution of wind patterns. PM₁₀ concentrations within a 2 km radius of the target area were significantly elevated, ranging from 4.0% to 74.8% of the annual average standard of 50 μg/m^3^, with absolute concentrations between 2 and 37.38 μg/m^3^. Chronic disease data were subsequently analyzed through cohort follow-up studies in Banwol-dong. The cohort analysis using National Health Insurance Service data revealed significant findings regarding the impact of PM₁₀ exposure on health outcomes. PM₁₀ concentrations were particularly high in 2019 and 2017, with the average concentration in 2017 (52.23 μg/m^3^) statistically significantly lower than in 2020 (67.70 μg/m^3^) (*P* = 0.0141). In the health analysis, exposure to higher PM₁₀ levels was associated with increased incidence of respiratory diseases, particularly acute lower respiratory infections and asthma. These findings are consistent with earlier studies conducted in other industrial regions of Korea. The slightly higher rhinitis incidence in the control area appears to reflect non-industrial factors such as allergen exposure, healthcare utilization patterns, and demographic differences rather than an effect of industrial pollution. Although the incidence of rhinitis was slightly higher in the control area than in the exposure area, this finding is likely attributable to non-industrial factors. Rhinitis is strongly associated with allergic and lifestyle-related conditions, such as pollen exposure, indoor environmental quality, smoking habits, and differences in healthcare utilization. Residents in the control area may have greater access to medical services or a higher tendency to seek medical attention, which can increase diagnosis rates independently of environmental exposure. Furthermore, some demographic and environmental characteristics of the two regions differ; for example, the control area includes more suburban residential zones with higher vegetation coverage, which may increase pollen-related allergic responses, whereas the exposure area is more industrialized with less greenery but higher levels of air pollutants. In contrast, the overall incidence of respiratory diseases, particularly acute lower respiratory infections and asthma, was significantly higher in the exposure group. This result reflects the chronic impact of industrial air pollution, including particulate matter and chemical emissions, which are more directly linked to long-term respiratory morbidity. Taken together, these findings suggest that the elevated rhinitis rates in the control area are influenced by background allergic or diagnostic factors, whereas the higher burden of respiratory diseases in the exposure area provides stronger evidence of the adverse health effects of industrial pollution. Regarding respiratory diseases, the incidence of acute upper respiratory tract infection was significantly higher in the exposed area, with 15,189 cases (57.05%) compared to 23,961 cases (52.83%) in the control area (p < 0.01). However, no significant difference was observed in other upper respiratory tract diseases between the two areas (p = 0.4510), with 74,950 (74.58%) cases in the exposed area and 128,434 (74.71%) in the control area. For acute lower respiratory tract infections, incidence was significantly higher in the exposed area, with 25,429 cases (47.03%) versus 36,108 cases (43.17%) in the control area (p < 0.01). Asthma incidence was also significantly elevated in the exposed area, with 129,659 cases (93.87%) compared to 200,437 cases (90.57%) in the control area (p < 0.01). These findings align with previous studies on respiratory health effects in Korean industrial complexes, confirming that conditions such as acute upper respiratory tract infections, asthma, and rhinitis are consistently observed among exposed populations. This underscores the urgent need for ongoing monitoring and regulation of pollutants in both workplace and residential areas to mitigate health risks associated with environmental exposure [28, 29]. However, the observed associations should be interpreted cautiously. Although elevated pollutant levels coincided with increased respiratory morbidity, the study design does not establish direct causality. Individual-level confounders, such as underlying health conditions, lifestyle factors, and the indirect effects of COVID-19 during the later study years, may have influenced the results. Nevertheless, the consistency of patterns across multiple data sources suggests a potential relationship between industrial emissions and respiratory health risks.

## Conclusions

This study suggests that long-term exposure to air pollutants in an industrial complex may be associated with increased respiratory disease burden among nearby residents. By linking environmental monitoring data with cohort follow-up information from the National Health Insurance Service, we were able to characterize exposure patterns and identify health outcomes that showed notable differences between exposed and control areas. Although this study does not prove causation, the findings offer valuable insights into potential chronic health risks related to industrial pollution. Importantly, this research contributes baseline information that can support future environmental health investigations and local public health planning. The approach used—combining dispersion modeling, spatial exposure classification, and long-term residential history—may be applicable to other industrial regions in Korea. This study has several limitations. First, exposure misclassification may have occurred because pollutant levels were estimated using annual averages rather than high-resolution temporal data. In addition, important confounding factors—such as socioeconomic status, underlying health conditions, and lifestyle characteristics including smoking—could not be fully controlled. The analysis focused primarily on PM₁₀, and other key pollutants such as VOCs, SO₂, NO₂, and heavy metals were not incorporated, which may limit the completeness of the exposure assessment. Health outcomes during the later study period may also have been influenced by COVID-19, particularly through changes in healthcare-seeking behavior. Furthermore, because the cohort was restricted to a single industrial region, the findings may not be fully generalizable to other areas. Lastly, correlation and time-series analyses were not conducted due to data constraints, limiting our ability to assess short-term fluctuations or temporal relationships between pollution levels and health outcomes. Despite these constraints, the methodological framework used here—particularly the incorporation of residential stability and long-term health records—provides a foundation for developing more refined environmental epidemiological studies. Continued monitoring and the establishment of expanded longitudinal cohorts will be essential for confirming these associations and guiding evidence-based policy responses.

## Supplementary Information

Supplementary Material 1.

## Data Availability

All the data generated, synthesized, and analyzed during the study are included in this review.
