# Supplementary material for: Air pollution and chronic disease in South Korea’s industrial zones: evidence from a cohort follow-up study
Source: BMC Public Health. 2026 Jun 18;26:2785. doi: 10.1186/s12889-026-27236-0 (PMC13637267; doi:10.1186/s12889-026-27236-0)
Supplement: Supplementary file 1 [file 12889_2026_27236_MOESM1_ESM.pdf]

위 원 회 심 의 결 과 서 Committee deliberation results

접수번호 (Registration Number)	2020-0069	IRB과제관리번호 (IRB Assignment Management Number)	CBNU-202005-BMSBBR-0069-01
승인일자 (Approval date)	2020년 05월 10일	승인유효일자 (Approval Effective Date)	2020년 12월 31일
심의신청종류 (Type of application for deliberation)	☑ 초기신규심의 ☐ 보완후 재심의 ☐ 연구계획변경심의 ☐ 지속심의 ☐ 연구중지보고심의 ☐ 기타		
심의종류 (Type of deliberation)	☐ 심의면제 ☑ 신속심의 ☐ 정식심의 ☐ 심의반려		
과제명 (Project Title)	국가산단지역 주민 환경오염 노출 및 건강영향조사(경기·충청, 3단계, 3차년도)		
소속	의과대학		
[illegible]	[illegible]		

Initial new review / Reconsideration after supplementation
Review of research plan changes / Continuing deliberation
Review of research suspension report / etc

Exemption from deliberation / **Expedited review** / formal deliberation / Refusal of deliberation

Environmental pollution exposure and health impact survey for residents of national industrial complexes (Gyeonggi-Chungcheong, 3rd stage, 3rd year)

belong : medical school

Research director : Yongdae Kim / **Researcher Choi kil-yong**

귀하께서 제출하신 연구계획서를 본 생명윤리심의위원회가 검토한 결과, 아래와 같이 결정되었습니다.

As a result of reviewing the research plan submitted by you, the Bioethics Review Committee has decided as follows.

심의결과 (Deliberation result)	☑ 승인 ☐ 시정승인 ☐ 보완 후 재심의 ☐ 반려 ☐ 심의면제 ☐ 조기종료 ☐ 일시정지 ☐ 승인된 연구 및 사항의 중지 또는 보류
지속심의일정 (Continuing deliberation schedule)	☐ 해당(다년도 과제는 차년별 지속심의 대상) ☑ 해당없음

approval / City administration approval / Reconsideration after supplementation
Companion / Exemption from deliberation / Early termination
Pause / Suspension or suspension of approved studies and matters

Applicable (multi-year projects are subject to continuous review each year) / **Not applicable**

※ 연구책임자는 본 위원회의 심의결과에 대하여 이의가 있을 경우, 심의결과 통지일로 부터 2주 이내에 서면으로 이의신청을 할 수 있습니다.

※ If the principal investigator has an objection to the deliberation results of this committee, he or she may file an objection in writing within two weeks from the date of notification of the deliberation results.

충북대학교 생명윤리심의위원회

Chungbuk National University Bioethics Review Committee

위원장 장석천
